# Supplementary material for: Identification of photoperiod-induced specific miRNAs in the adrenal glands of Sunite sheep (Ovis aries)
Source: Front Vet Sci. 2022 Jul 22;9:888207. doi: 10.3389/fvets.2022.888207 (PMC9354845; doi:10.3389/fvets.2022.888207)
Supplement: Supplementary file 1 [file Data_Sheet_1.ZIP › Supplementary file/miRDeep2 file.zip!/miRDeep2 file/Know_Structure/SPLP42A3/oar-mir-495.pdf]

miRBase precursor : oar-mir-495
Total read count : 25374
oar-miR-495-5p read count : 58
oar-miR-495-3p read count : 25301
remaining reads : 15

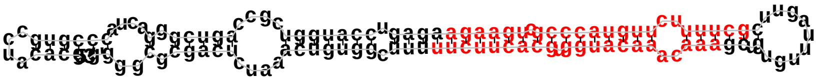

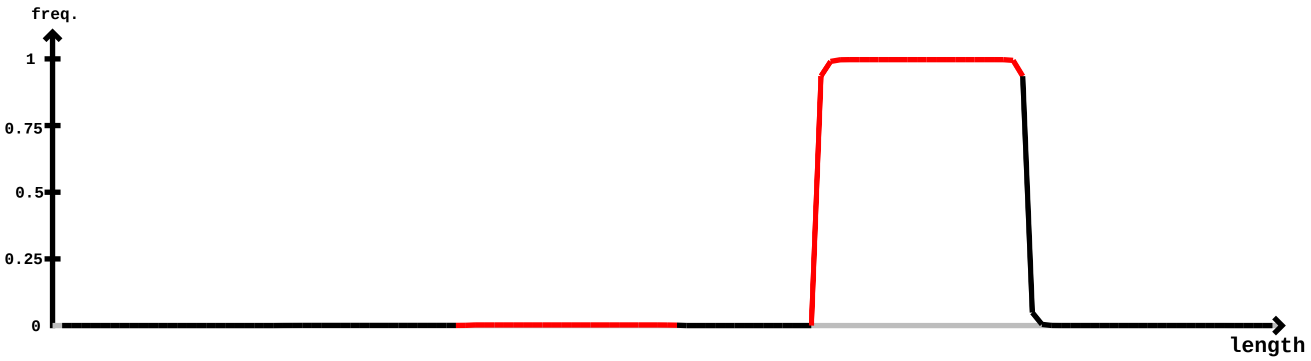

oar-miR-495-3p

oar-miR-495-5p

```
5'- gcacauccgugcccaucagggcugaccgcugguaccugagaagaagucgcccauguucuuuucgcuugauuugugucgaaacaaacauggugcacuucuuuuucggugucaaaucucagcgcgggg  -3'   exp
    ((((....))))((....(.(((((....(((((((.((((((((((.(((((((((..((((((.........).)))))..))))))).))))))))))))).)))))))....))))).)..))   reads   mm
    ....................gcugaccgcugguaccugagaa....................................................................................   1       0
    ......................ugaccgcugguaccugaga.....................................................................................   2       0
    ......................ugaccgcugguaccugagaa....................................................................................   5       0
    .......................gaccgcugguaccugagaa....................................................................................   2       0
    ........................accgcugguaccugagaa....................................................................................   2       0
    .........................ccgcugguaccugagaa....................................................................................   1       0
    .........................................agaagucgcccauguucuuuu................................................................   6       0
    .........................................agaagucgcccauguucuuuuc...............................................................   1       0
    .........................................agaagucgcccauguucuuuucg..............................................................   2       0
    ..........................................gaagucgcccauguucuuuu................................................................   2       0
    ..........................................gaagucgcccauguucuuuuc...............................................................   14      0
    ..........................................gaagucgcccauguucuuuucg..............................................................   33      0
    ..............................................................................aaacaaacauggugcacuu.............................   2       0
    ..............................................................................aaacaaacauggugcacuuc............................   62      0
    ..............................................................................aaacaaacauggugcacuucu...........................   1399    0
    ..............................................................................aaacaaacauggugcacuucuu..........................   21496   0
    ..............................................................................aaacaaacauggugcacuucuuu.........................   762     0
    ..............................................................................aaacaaacauggugcacuucuuuu........................   16      0
    ...............................................................................aacaaacauggugcacuuc............................   4       0
    ...............................................................................aacaaacauggugcacuucu...........................   104     0
    ...............................................................................aacaaacauggugcacuucuu..........................   941     0
    ...............................................................................aacaaacauggugcacuucuuu.........................   329     0
    ...............................................................................aacaaacauggugcacuucuuuu........................   19      0
    ................................................................................acaaacauggugcacuucu...........................   3       0
    ................................................................................acaaacauggugcacuucuu..........................   49      0
    ................................................................................acaaacauggugcacuucuuu.........................   33      0
    ................................................................................acaaacauggugcacuucuuuu........................   65      0
    ................................................................................acaaacauggugcacuucuuuuu.......................   4       0
    .................................................................................caaacauggugcacuucuu..........................   3       0
    .................................................................................caaacauggugcacuucuuu.........................   2       0
    .................................................................................caaacauggugcacuucuuuu........................   4       0
    .................................................................................caaacauggugcacuucuuuuu.......................   3       0
    ..................................................................................aaacauggugcacuucuu..........................   1       0
    ....................................................................................................uuucggugucaaaucucagcg.....   2       0
```

oar-miR-495-3p

oar-miR-495-5p

gcacauccgugcccaucagggcugaccgcugguaccugagaagaagucgcccauguucuuuucgcuugauuugugucgaaacaaacauggugcacuucuuuuucggugucaaaucucagcgcgggg
